# Model-Based Optimisation of Deferoxamine Chelation Therapy

**DOI:** 10.1007/s11095-015-1805-0

**Published:** 2015-11-10

**Authors:** Francesco Bellanti, Giovanni C. Del Vecchio, Maria C. Putti, Carlo Cosmi, Ilaria Fotzi, Suruchi D. Bakshi, Meindert Danhof, Oscar Della Pasqua

**Affiliations:** Division of Pharmacology, Leiden Academic Centre for Drug Research, Leiden, The Netherlands; Clinical Pharmacology & Therapeutics, University College London, BMA House, Tavistock Square, WC1H 9JP London, UK; Clinical Pharmacology Modelling & Simulation, GlaxoSmithKline, Stockley Park, Uxbridge, UK; Azienda Ospedaliera Universitaria Consorziale, Policlinico di Bari, Bari, Italy; Azienda Ospedaliera di Padova, Clinica di Oncoematologia Pediatrica, Padova, Italy; Azienda Ospedaliera Universitaria, Policlinico di Sassari,, Sassari, Italy

**Keywords:** adherence, deferoxamine, disease modelling, dose rationale, iron overload, PKPD modelling

## Abstract

**Purpose:**

Here we show how a model-based approach may be used to provide further insight into the role of clinical and demographic covariates on the progression of iron overload. The therapeutic effect of deferoxamine is used to illustrate the application of disease modelling as a means to characterising treatment response in individual patients.

**Methods:**

Serum ferritin, demographic characteristics and individual treatment data from clinical routine practice on 27 patients affected by β-thalassaemia major were used for the purposes of this analysis. The time course of serum ferritin was described by a hierarchical nonlinear mixed effects model, in which compliance was parameterised as a covariate factor. Modelling and simulation procedures were implemented in NONMEM (7.2.0).

**Results:**

A turnover model best described serum ferritin changes over time, with the effect of blood transfusions introduced on the ferritin conversion rate and the effect of deferoxamine on the elimination parameter (Kout) in a proportional manner. The results of the simulations showed that poor quality of execution is preferable over drug holidays; and that independently of the compliance pattern, the therapeutic intervention is not effective if >60% of the doses are missed.

**Conclusions:**

Modelling of ferritin response enables characterisation of the dynamics of iron overload due to chronic transfusion. The approach can be used to support decision making in clinical practice, including personalisation of the dose for existing and novel chelating agents.

**Electronic supplementary material:**

The online version of this article (doi:10.1007/s11095-015-1805-0) contains supplementary material, which is available to authorised users.

## Introduction

### Transfusional Iron Overload

Beta-thalassaemia major is a hereditary blood disorder and patients affected by this disease require regular red blood cell (RBC) transfusions to survive (1–7). Without the chronic transfusion regimen, patients would die before the third decade of life (2,4,5,7,8). Even though a significant improvement has been achieved in the management of the chronic transfusion regimen in the past decades, the therapy will eventually lead to a series of complications. Iron overload is the most common and relevant one and it is associated with several co-morbidities such as cardiac dysfunction, liver fibrosis, hypogonadism, hypothyroidism, hypoparathyroidism and diabetes mellitus (6,9,10). Cardiac disease caused by myocardial siderosis is the most relevant complication, causing death in 71% of the patients affected by transfusion-dependent diseases (11).

For a complex process such as iron overload, understanding of the dynamics of the disease and its progression is crucial to adequately evaluate the impact of a therapeutic intervention. This complexity is characterised also by the fact that the biomarker ferritin does not provide a direct link between total body or tissue iron accumulation at specific time points. The absence of such a relationship is partly explained by the influence of other pathological mechanisms (e.g., inflammatory disorders, and/or liver status) which can affect the iron interchange between organs and the circulatory system (2,12–14). On the other hand, changes in ferritin levels are still helpful for the management of the disease and maintaining serum ferritin below a threshold of about 2500 μg/L is a widely accepted therapeutic goal (2,3,5–7). Nonetheless, several clinical questions are not yet fully explored, *e.g.,* how much time is required in order to observe a true response, or in order to reach clinically safe serum ferritin levels.

### Iron Chelation Therapy with Deferoxamine

Given that the ability of our body to remove the excess of iron is limited to a maximum of 1–2 mg/day (*e.g.*, loss of intestinal cells), treatment with iron chelators is vital to prevent its accumulation and related complications (15–18). In this analysis we are mainly interested in the iron chelating agent deferoxamine. Deferoxamine was the first iron chelator approved for human use and has been available for the treatment of iron overload for more than 35 years (2,6,15–19). It is an exadentate chelator that binds iron in a 1:1 ratio. The drug is rapidly absorbed after intramuscular and subcutaneous administration, but it cannot be absorbed orally. Several dosing regimens and routes of administration have been proposed and used in the past for deferoxamine in patients affected by transfusion-dependent haemoglobinopathies but in the majority of cases it is given as an 8 to 12 h nightly subcutaneous infusion (5 to 7 days a week) (2,19–21). The serum protein binding is less than 10% and the drug undergoes the following metabolic reactions: transamination and oxidation; beta-oxidation; decarboxylation and N-hydroxylation. The average recommended daily dose lies between 20 and 60 mg/kg and in patients with haemochromatosis the drug has an half-life of 5.6 h (20–22).

Deferoxamine binds free iron by preventing the uptake of NTBI (Non-Transferrin Bound Iron) into organs but it also acts within the cell where it enters by endocytosis, stimulates the degradation of ferritin *via* the lysosomes and subsequently binds the realeased iron. The iron bound to deferoxamine is then excreted in urine and feces (2,6,21,23).

Despite the availability of new oral iron chelators and several limitations regarding the use of deferoxamine, such as compliance issues due to the parenteral administration, inadequate cardiac iron removal and auditory, ocular and neurological toxicities (6,16,18,19,24), deferoxamine is still the most common used therapy for the treatment of iron overload.

The current investigation focuses on the use of a model-based approach to gain insight into key factors that play a role in iron overload, with the objective of quantifying the therapeutic effect of deferoxamine and characterising the role of relevant covariates on the underlying disease progression. Furthermore, we show how modelling and simulation (M&S) can be applied to support decision making in clinical practice providing a framework to predict changes in the disease status and the evaluate the impact of predefined therapeutic regimens.

## Methods

### Data

The data analysis was performed using retrospective clinical data from three different Italian centres: A.O. Universitaria Consorziale Policlinico di Bari U.O. Pediatria Federico Vecchio; A.O. Universitaria Policlinico di Sassari Clinica Pediatrica, ASL 1 D.H. Per Talassemia; A.O. di Padova Clinica di Oncoematologia Pediatrica. The study has been conducted in full conformance with the principles of the Declaration of Helsinki and with the local laws and regulations concerning clinical trials. The protocol and the informed consent documents have been formally approved by the relevant research ethics committee of each clinical site.

Clinical data were collected retrospectively for a maximum of 10 years from 27 patients affected by transfusion-dependent diseases, receiving deferoxamine as monotherapy for iron chelation. Patients were followed according to standard clinical practice, which includes case specific dose adjustments. Modifications to the dosing regimen were recorded and transcribed into the analysis dataset; the most prevalent dose was 40 mg/kg. Baseline characteristics of the patient population are provided in Table I. Serum ferritin was the main endpoint of interest and was measured every 2 to 3 months; patients contributed with 40.2 observations on average (sd: 17), with a minimum of 4 samples per year.

**Table I:** Baseline Characteristics of the Patient Population (*n* = 27) Included in the Analysis

	Units	Median	Range
Age	Years	14.6	6.8–19.9
Weight	kg	46	17.5–71
Height	cm	154	111–173
TSH	mIU/L	2.34	0.58–83.2
FT4	ng/dL	1.05	0.73–1.43
AST	U/L	33	7–159
ALT	U/L	56	9–372
Glucose	mg/dL	91	52–444
Creatinine	mg/dL	0.6	0.2–1.12
Ejection fraction	%	64	35–77
Ferritin	μg/L	2260	393–8500

### PK Model of Deferoxamine

**Fig. 1:**
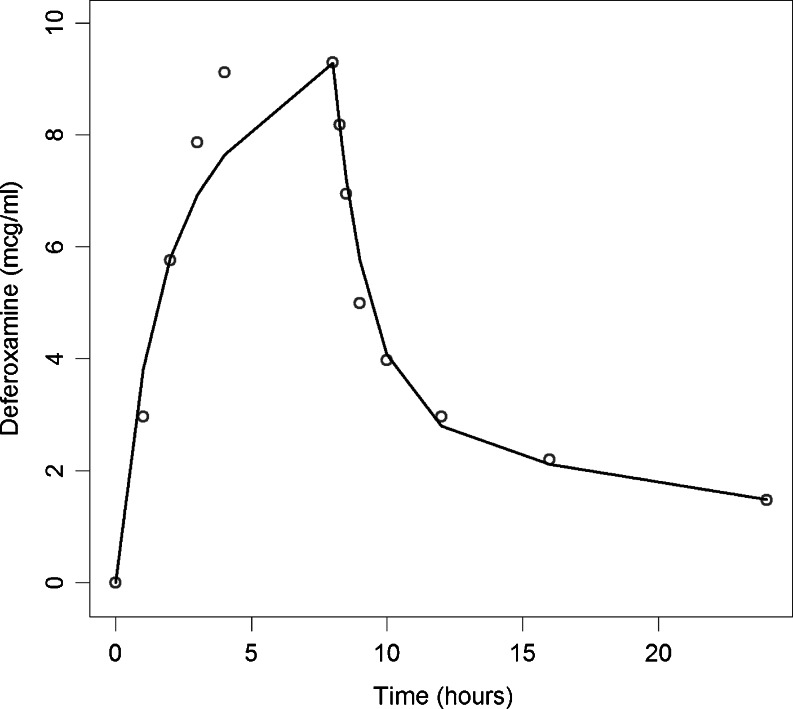
Predicted and observed pharmacokinetic profile of deferoxamine. The *circles* represent the mean deferoxamine concentrations reported in literature (25). The *solid line* represents the population model prediction.

A two compartment pharmacokinetic model with zero-order absorption (8 h subcutaneous infusion) and first-order elimination processes was used to describe the time course of deferoxamine in plasma and subsequently derive the average steady state concentration (Css^AV^) for the population of interest. Deferoxamine plasma levels were based on the dosing regimen information collected from the patients' medical history at the clinical centres. The model was built using literature data (25) by fitting a mean pharmacokinetic profile in adult patients affected by transfusion-dependent haemoglobinopathies receiving a 40 mg/kg dose of deferoxamine as an 8 h subcutaneous infusion. The final PK parameters obtained as input for the data analysis included apparent clearance (19.3 L/h), intercompartmental clearance (17.6 L/h), apparent volume of distribution (77.4 L) and apparent peripheral volume of distribution (238.0 L). Subsequently fixed allometric scaling (exponent of 0.75 on apparent clearances and 1.00 on the apparent volumes of distribution) was used to extrapolate Css^AV^ in adolescents and children. Population prediction (PRED) were used to generate Css^AV^ values in the population of interest. The fitting of the model is shown in Fig. 1.

### Disease Model of Iron Overload

A disease model of iron overload in patients affected by transfusion-dependent diseases was used to account for the effect of blood transfusions on serum ferritin levels. It consisted of an indirect response model where the basal turnover of ferritin is depicted by a zero-order production rate (Kin) and a first-order degradation rate (Kout) and the parameter describing the disease component is represented by an additive production rate (CRT) triggered by the transfusion regimen, which was found to be non-linearly correlated to the disease status (actual ferritin levels).

1 $$ \frac{dFERRITIN}{dt}= Kin+CRT- Kout\times FERRITIN $$

2 $$ CRT=SCL\times {e}^{-SHP\times FERRITIN} $$

where SCL is a scaling factor and SHP is the shape factor of the correlation between the disease status and the production rate (CRT). The population parameters of the disease model were kept fixed when performing the retrospective PKPD analysis of the clinical data.

### PKPD Modelling of Deferoxamine

The software R (v.2.14.0) (26) was used for statistical summaries as well as data manipulation and preparation for modelling purposes. Nonlinear mixed effects modelling was performed in NONMEM version 7.2 (Icon Development Solutions, USA).

Model building criteria included: (i) successful minimisation, (ii) standard error of estimates, (iii) number of significant digits, (iv) termination of the covariance step, (v) correlation between model parameters and (vi) acceptable gradients at the last iteration. Comparison of nested hierarchical models was based on the likelihood ratio test (LRT). Goodness of fit was assessed by graphical methods, including population and individual predicted *vs.* observed concentrations, conditional weighted residuals *vs.* observed concentrations and time, correlation matrix for fixed *vs.* random effects, correlation matrix between parameters and covariates and normalised predictive distribution error (NPDE) (27).

Fixed and random effects were introduced into the model in a stepwise manner. Inter-individual variability and inter occasion variability (IOV) in the model parameters was assumed to be log-normally distributed. A parameter value of an individual *i* (post hoc value) is therefore given by the following equation:

$$ {\uptheta}_{\mathrm{i}}={\uptheta}_{\mathrm{TV}}\ast {e}^{\left(\upeta \mathrm{i}+\mathrm{IOVi}\right)} $$

in which θ_TV_ is the typical value of the parameter in the population and η_i_ and IOV_i_ are assumed to be random variables with zero mean and variance ω^2^. Residual variability, which comprises measurement and model error, was described by a proportional error model. This means that for the j^th^ observed concentration of the i^th^ individual, the following relation applies:

$$ {\mathrm{Y}}_{\mathrm{ij}}={\mathrm{F}}_{\mathrm{ij}}+{\upvarepsilon}_{\mathrm{ij}}\ast W $$

where F_ij_ is the predicted concentration, ε_ij_ is a random variable with mean zero and variance σ^2^, and W is a proportional weighing factor for ε.

Different concentration-effect relationships (*e.g.*, Emax model, linear model, *etc.*) were tested on the disease model presented in Eq. 1 to characterise the effect of deferoxamine on serum ferritin levels. Deferoxamine average steady-state concentrations (Css^AV^) generated with the PK model described above were used as a measure of drug exposure. The effect of deferoxamine (DFO) was introduced in a proportional way on the degradation rate (Kout) of ferritin as shown in Eq. 3 which is derived from Eq. 1.

3 $$ \frac{dFERRITIN}{dt}= Kin+CRT- Kout\times FERRITIN\times \left(1+DFO\right) $$

4 $$ DFO=SLP\times SCs{s}^{AV} $$

where DFO is the effect of deferoxamine on the degradation rate (Kout) of the disease model, SLP is the slope parameter of the concentration-effect relationship, and SCss^AV^ are the steady state concentrations simulated with the deferoxamine PK model.

In addition, two disease model parameters, namely, the scaling (SCL) and the shape (SHP) factors presented in Eq. 2 were found to be non-linearly correlated with the actual disease status according to the following relationships:

5 $$ SC{L}_i=SC{L}_{ref}\times {\left(\frac{ FERRITI N}{FERRITI{N}_{med}}\right)}^{\theta x} $$

6 $$ SH{P}_i=SH{P}_{ref}\times {\left(\frac{ FERRITI N}{FERRITI{N}_{med}}\right)}^{\theta x} $$

where SCL_ref_ and SHP_ref_ are the reference parameters in the population of interest, SCL_i_ and SHP_i_ are the individual parameters, FERRITIN_med_ is the median ferritin value in the population of interest and θx is the estimated exponent of the relationship.

The validation of the final model was based on graphical and statistical methods, including visual predictive checks (28). Bootstrap (1000 samples) was used to identify bias, stability and accuracy of the parameter estimates (standard errors and confidence intervals). The bootstrap procedures were performed in PsN v3.5.3 (University of Uppsala, Sweden) (29), which automatically generates a series of new data sets by sampling individuals with replacement from the original data pool, fitting the model to each new data set.

### Evaluation of the Role of Compliance

At an early stage of the model building phase model misspecification was observed, as the model could not appropriately describe the data (See supplemental material, Figure 1S) based on the assumption that the population under investigation as one single population. In fact, two different ferritin profiles were identified and patients were initially considered as responders and non-responders. The responders showed very stable profiles around 2500 μg/L serum ferritin, whereas the non-responders showed very steep increases in ferritin levels and appeared not to be able to return to a less severe state of the disease. A mixture model improved the quality of the fitting but would still not allow an adequate characterisation of the individual profiles. Demographic factors were compared among the two sub-populations and no significant differences were identified that could justify the difference in response to treatment.

We have therefore decided to investigate the possible mechanisms responsible for the different response profiles using the information available in the literature. Treatment compliance was found to be the major cause of these differences. In the papers by Gabutti *et al.* (30) and Galanello *et al.* (31) serum ferritin profiles are quite stable over the observational period (See Fig. 1 and supplemental material Figure 2S) as in our responder group and compliance is in both cases higher than 95%. In other investigations (20,30,32) Kaplan-Meier analyses show the relationship between survival and treatment compliance, providing evidence of the fact that poor adherence has a major impact on response. In particular the work by Olivieri *et al.* (32) shows how survival can directly be linked to the observed ferritin levels.

The absence of quantitative data on treatment compliance in our retrospective study did not allow us to directly use this variable to account for such differences and was a clear obstacle for the analysis. To overcome this limitation we used the work carried out by Olivieri *et al.* (32) as a reference and derived a new variable (CMPL) based on the percentage of observations for each patient above the threshold of 2500 μg/L ferritin. CMPL had a median value of 21% (range of 0–100) and mean of 32% (95% CI: 0.9–98%) in the population under investigation. The new variable (CMPL) was introduced into the model as follows:

7 $$ DFO=SLP\times T{C}_{ss}^{AV} $$

8 $$ T{C}_{ss}^{AV}=S{C}_{ss}^{AV}\times \left(1- CMPL\right) $$

where DFO is the effect of deferoxamine, SLP is the slope parameter of the concentration-effect relationship, and TCss^AV^ is the “true” steady state concentration after accounting for the impact of treatment compliance (CMPL). TCss^AV^ values are derived from the simulated steady state concentrations (SCss^AV^) corrected for treatment compliance as shown in Eq. 7.

**Fig. 2:**
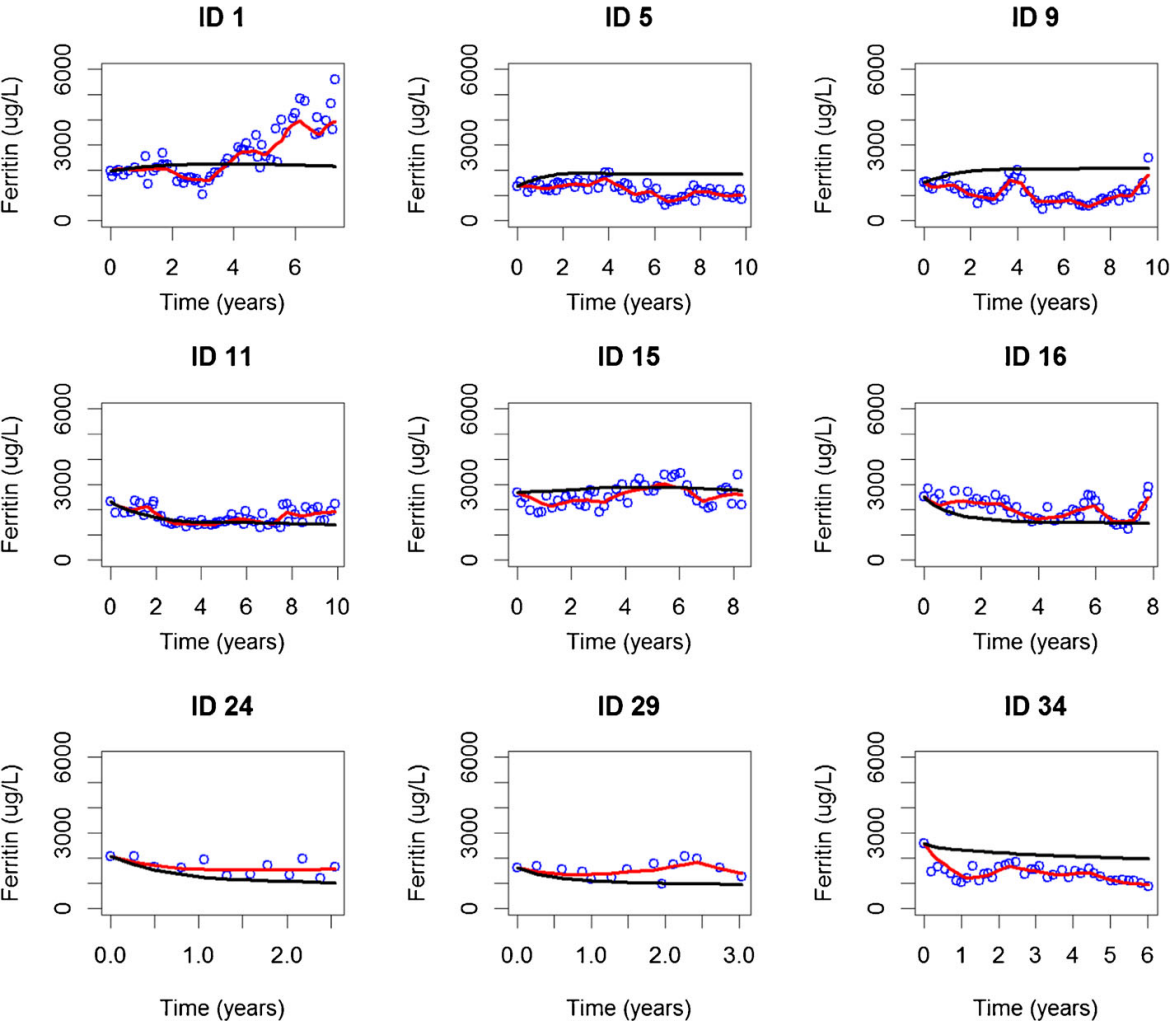
Individual plots of 9 randomly selected patients: observed data are shown as *blue circles*; the *black and red solid lines* represent, respectively, the population (Pred) and individual predictions (IPred).

The incorporation of treatment compliance into the model provided a significant improvement in the fitting of the data (as shown in Fig. 2 and supplemental material Figure 2S) and allowed a more accurate description of the therapeutic intervention.

### Simulation Scenarios (Decision-Making in Clinical Practice)

Simulations were performed to investigate the impact of different exposure levels and various compliance scenarios on the clinical response, as assessed by serum ferritin levels. Time to reach 2500 μg/L serum ferritin (threshold between moderate and severe iron overload (2,6,20,32,33)) was chosen as a comparison measure between different scenarios. The differential equation solver ode15s from the software MATLAB (version R2010b) (34) was used for the simulations, whereas the software R (v.2.14.0) (26) was used for graphical summaries. The ode15s, which is a multistep solver and uses numerical differentiation formulas, is particularly suitable for stiff systems (35,36).

**Fig. 3:**
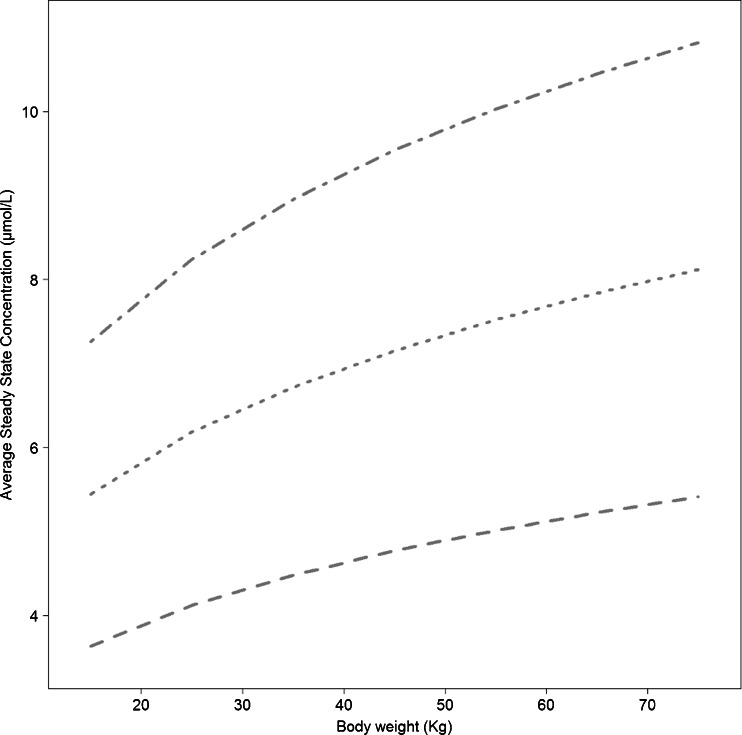
Average steady state concentration by body weight for 7 typical individuals receiving three different deferoxamine doses: 30 (- - -), 45 (....) and 60 (-.-.-) mg/kg/day.

Three dosing regimens (30, 45 and 60 mg/kg/day for 5 days a week) were used to generate Css^AV^ in a patient population with body weight ranging from 15 to 75 kg. A visual representation of the Css^AV^ by body weight for the three dosing regimens can be found in Fig. 3. This enabled the assessment of the impact of different exposures on the endpoint of interest (time to reach the threshold). Each exposure level was then tested on patients starting at different baselines ranging from 3000 to 12,000 μg/L serum ferritin. In this set of simulations the exposure was assumed to be constant over time.

In addition, simulation scenarios were evaluated to characterise the impact of various compliance scenarios on changes in ferritin levels. Our main interest in this case was to show how a drug-disease model can be used prospectively in clinical practice to predict individual patient response. Given the large number of scenarios that were evaluated here, the discussion and presentation of the results will be limited to the case in which a virtual patient of 45 kg receives 45 mg/kg/day deferoxamine 5 days per week. The different scenarios investigated are also presented in Table II, where compliance is stratified into four groups: 1 year, 6 months, 2 months and 1 month. Compliance patterns in each group were further clustered into two categories, namely 1) poor quality of execution, i.e., missed single doses at random, and 2) drug holidays, i.e., doses that are missed consecutively during a given period of time. For example, in the poor quality of execution scenario, missing 40% of the doses at random means that 100 single doses were missed randomly over a period of 1 year; whereas in the drug holidays scenario, missing 40% of the doses means missing 100 consecutive doses over a period of 1 year (*i.e.,* the first 100 doses are missed and the remaining 150 are taken). The iterations were stopped if more than 5 years were needed to reach the threshold of 2500 μg/L serum ferritin. Five years were considered as a clinically relevant time limit for the purposes of the analysis. Patients reaching the predefined ferritin threshold in more than 5 years do not influence the results of the analysis or the comparison of the treatment response for the different dosing regimens and compliance patterns.

**Table II:** Simulation Scenarios for the Evaluation of Different Compliance Levels. Full Adherence is Equivalent to 250 Doses Per Year

	% of missed doses	Number of missed doses during the stratification period				
		Missed single doses at random	Missed consecutive doses (*i.e.*, drug holidays)			
		Stratification				
		1 year	1 year	6 months	2 months	1 month
Scenario 1	10%	25	25	/	5	/
Scenario 2	20%	50	50	25	10	5
Scenario 3	30%	75	75	/	15	/
Scenario 4	40%	100	100	50	20	10
Scenario 5	50%	125	125	/	25	/
Scenario 6	60%	150	150	75	30	15
Scenario 7	70%	175	175	/	35	/
Scenario 8	80%	200	200	100	40	20
Scenario 9	90%	225	225	/	45	/

## Results

### Disease Model

As described in the methods a disease model was previously developed for iron overload [unpublished results] and its performance in describing the impact of blood transfusions on serum ferritin was confirmed in this analysis. The effect of blood transfusions was introduced as a conversion rate on the production rate of ferritin, which was found to be inversely correlated with the disease status, as shown in Eqs. 1 and 2. In addition, two disease model parameters, namely, the scaling (SCL) and the shape (SHP) factors presented in Eq. 2 were found to be non-linearly correlated with the actual disease status. Their inclusion in the model provided a significant decrease in the objective function value (OFV) and allowed a better description of the data. Further improvement was obtained after the inclusion of inter-occasion variability (IOV = 57.4%) on the conversion rate, which resulted in a significant drop in the OFV (Δ 443) allowing a better description of the individual profiles.

### Drug Model

The effect of deferoxamine (DFO) was introduced in a proportional way on the degradation rate (Kout) of ferritin. A linear model was preferred to a non-linear model (*i.e.,* Emax) given the fact that it accurately described the changes in ferritin observed clinically. The limited range of doses and number of patients did not provide sufficient information to support the characterisation of a non-linear model. Furthermore, the implementation of treatment compliance as a factor on the exposure of deferoxamine improved considerably the quality of the fitting and the model performance. The inclusion of inter-individual variability (IIV) on the slope parameter also reduced significantly the OFV and improved goodness of fit and visual predictive check (VPC) diagnostics. An overview of the final model parameters and bootstrap results is presented in Table III.

**Table III:** Parameter Estimates of the PKPD Model of Deferoxamine

Parameter	Estimate	Bootstrap (mean)	CV (%)
Kin (μg/h)	0.0002 (FIX)	/	/
Kout (h^−1^)	0.0000045 (FIX)	/	/
SHP (h^−1^)	0.00026 (FIX)	/	/
SCL (μg/h)	0.383 (FIX)	/	/
Slope (μg/conc)	4.81	5.16	15.7
Error proportional	−0.173	−0.17	6.5
DIS exp on SHP	1.29	1.08	57.4
DIS exp on SCL	0.845	0.67	51.9
IIV on Slope	0.082	0.105	80.9
IOV on CRT	0.252	0.29	43.1

*CRT* production rate due to transfusion regimen, *SHP* shape factor, *SCL* scaling factor, *DIS* disease status, *IIV* interindividual variability, *IOV* inter-occasion variability, *CV* coefficient of variation, *Bootstrap* 1000 samples

**Fig. 4:**
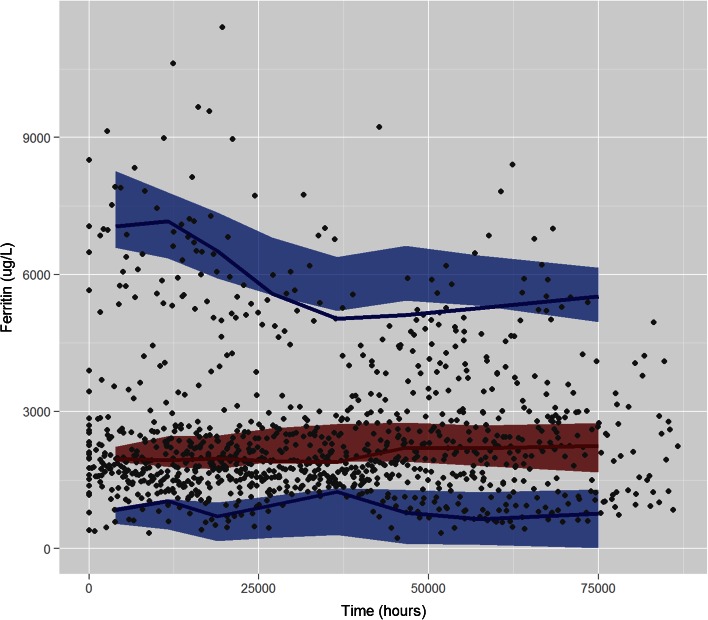
Visual predictive check: observed data are depicted by *grey circles*; the *red and blue solid line* represent, respectively, the median and the 5th and 95th percentiles of the observed data. The *red and blue shaded area* represent, respectively, the 95th CI of the median and the 95th CI of the 5th and 95th percentiles of the simulated data.

Internal model validation diagnostics were satisfactory. Individual predicted profiles and goodness-of-fit plots, as shown in Fig. 2 and supplemental material figures 2S along with VPC results (Fig. 4) reveal that the model provides an adequate and unbiased description of the data. In addition, NPDE summaries (figure 3S) show that the discrepancy between predicted and observed values can be assumed to be normally distributed.

### Simulation Scenarios (Decision-Making in Clinical Practice)

**Fig. 5:**
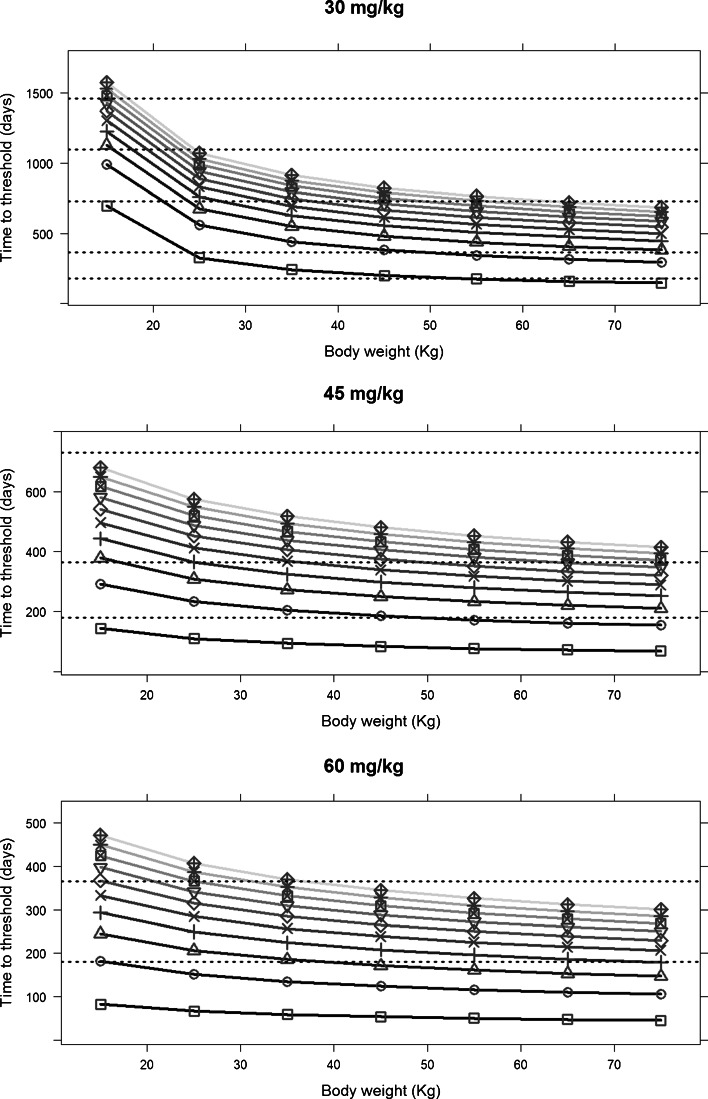
Time to reach a serum ferritin threshold of 2500 ug/L for varying exposure levels in patients with different body weights (15 to 75 kg). The panels show three scenarios where 30, 45 and 60 mg/kg dosing regimen were evaluated. Each *line* represents a different starting baseline ferritin level (*darker* to *lighter* shows an increase in the starting baseline levels). *Square*, *circle*, *triangle with point up*, *plus*, *cross*, *diamond*, *triangle with point down*, *square cross*, *star* and *diamond* plus represent starting baseline ferritin values, respectively of 3000, 4000, 5000, 6000, 7000, 8000, 9000, 10,000, 11,000 and 12,000 ug/L. The *dashed horizontal lines* represent indicative thresholds at 6 months, 1, 2, 3, 4 and 5 years.

The impact of different exposure levels and varying compliance levels on the clinical response, as assessed by serum ferritin levels was evaluated through model simulations. A summary of the results stratified by body weight and ferritin levels at the start of treatment is presented in Fig. 5 for the following dosing regimens: 30, 45 and 60 mg/kg/day for 5 days a week. Results clearly show that an appropriate clinical response cannot be achieved without adequate exposure to the chelating agent. The model also provides the opportunity to evaluate *a priori* the most suitable dosing regimen to achieve a desired therapeutic goal.

**Fig. 6:**
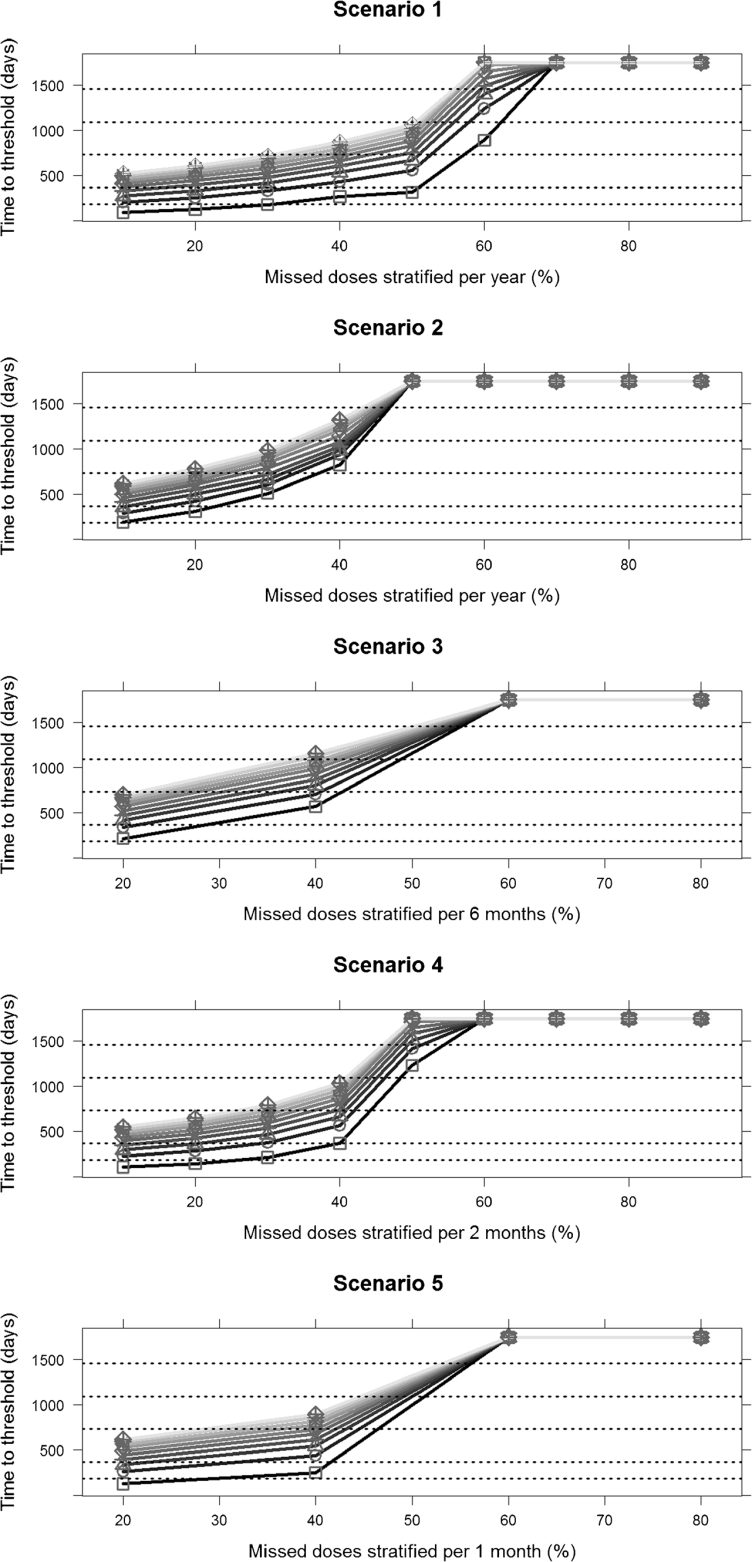
Time to reach a serum ferritin threshold of 2500 mcg/L for different compliance scenarios (10 to 90% of missed doses). The panels show five scenarios where different compliance patterns have been evaluated (see Table II). Each *line* represents a different starting baseline ferritin level (*darker* to *lighter* shows an increase in the starting baseline levels). *Square*, *circle*, *triangle with point up*, *plus*, *cross*, *diamond*, *triangle with point down*, *square cross*, *star* and *diamond* plus represent a starting baseline ferritin level, respectively of 3000, 4000, 5000, 6000, 7000, 8000, 9000, 10,000, 11,000 and 12,000 ug/L. The *dashed horizontal lines* represent indicative thresholds at 6 months, 1, 2, 3, 4 and 5 years.

Finally, we have investigated the impact of different compliance patterns on the time to reach the threshold of 2500 μg/L serum ferritin. Whereas simulations refer to one virtual patient of 45 kg receiving 45 mg/kg/day deferoxamine 5 days per week, several conclusions can be derived from these results: 1) a better and faster response is achieved if single doses are missed at random (reflecting poor quality of execution) (Fig. 6—scenario 1) as compared to doses missed consecutively (drug holidays) (Fig. 6—scenario 2) over a period of 1 year; 2) if doses are missed consecutively over a given period of time, the shorter the period the better the clinical response (as shown in Fig. 6—scenarios 2 to 5); 3) in all the scenarios, if more than 60% of the doses are missed (treatment compliance is lower than 40%) the therapeutic intervention is not effective; and finally 4) a reduction in treatment compliance, especially when moving from 30 to 60% of missed doses clearly leads to a significantly slower response. These findings indicate that even if the desired therapeutic outcome can be achieved the time to reach the proposed threshold might not be sustainable for the patient.

## Discussion

A model-based approach is proposed here as a tool for the evaluation of the impact of disease-, patient- and drug specific factors on iron chelation therapy in patients affected by transfusion-dependent haemoglobinopathies. The complexity of iron homeostasis and more specifically the ferritin response to chelation therapy requires an integrated approach that allows exploring the dynamics of iron overload and its progression. A drug-disease model was successfully developed and validated as shown in Table III and supplemental figures 2S and 3S. The analysis reveals a strong effect of the disease status on the overall iron/ferritin conversion rate, and highlights the role of treatment (drug exposure and compliance patterns) on the overall response and disease progression.

Simulation scenarios were used to investigate the impact of different exposure levels as well as the effect of varying patterns of compliance on serum ferritin levels. Results show that inadequate iron chelation therapy with sub-therapeutic exposure (Fig. 5) as well as poor adherence to the assigned dosing regimen (Fig. 6) significantly increase the time required to achieve a desired clinical response, and in some cases (*e.g.*, with treatment compliance lower than 40%) the therapeutic goal cannot be achieved at all. Even though these results might seem rather obvious, we are aware that clinically relevant changes in serum ferritin levels take place over a somewhat long period of time, and often crucial decisions have to be made before the clinical evidence is available. Our approach offers an opportunity to explore different scenarios before medical decisions have to be made. For example, the use of simulation scenarios may provide insight into whether lowering the dose (*i.e.*, a trade-off for a possible reduction in acute side effects) and an increased time to achieve the therapeutic goal would be sustainable for the patient. In the same way, given the importance of treatment compliance for a drug such as deferoxamine, the evaluation of simulation scenarios allows a more quantitative evaluation of the impact of different compliance patterns. This information can be used to support the decision making and to optimise the therapeutic intervention.

### Limitations

Some limitations must be considered in the context of this analysis. First of all, we developed a PK model based on literature data, which allowed us to use fixed allometric scaling, including mean population data and individual information. A more structured analysis of the PK of deferoxamine would reduce the uncertainty around the simulated exposure and explain part of the variability in PK that propagates into the pharmacodynamic parameter estimates. On the other hand, the proposed approach allowed us to characterise differences in the pharmacokinetics that could not have been evaluated based only on information about the dosing regimen; *e.g.*, changes in size are accounted for by allometric scaling.

A second aspect is the role of treatment compliance. The lack of quantitative data on adherence in the population under investigation was a clear impediment for the analysis. To overcome this issue we used the observed data to generate a variable that captures differences in compliance. This decision was supported by the information available in the literature; we found clear evidence that high compliance leads to stable ferritin levels over time and that poor adherence to deferoxamine therapy is strongly correlated with poor clinical outcome, as nicely depicted in the work by Gabutti *et al.* (30) (Kaplan-Meier analysis presented in Fig. 2). This was confirmed by a few other publications (20,31,32,37) and gave us the confidence that the approach taken here would be robust enough for the purposes of this analysis. In addition, we should emphasise that whilst not all levels of compliance have been tested, linear interpolation between scenarios does not affect the overall conclusions that can be drawn from the simulation results. For instance, the impact of 50% compliance can be inferred from data showing response for patients with 40 and 60% missed doses (see Fig. 6). Of course, additional simulations would need to be performed to obtain accurate figures for this scenario.

## Conclusion

In conclusion, modelling of ferritin response enables characterisation of the dynamics of iron overload in patients receiving chronic blood transfusions. Of note is the possibility to predict the time to achieve clinically safe levels of ferritin in individual patients. The approach can be used to support decision making in clinical practice, including personalisation of the dose for existing and novel chelating agents. Bearing in mind the limitations discussed above and the relative level of uncertainty, this model can be expanded to other transfusion-dependent haemoglobinopathies.

## Electronic supplementary material

Figure 1S (PDF 140 kb)

Figure 2S (PDF 152 kb)

Figure 3S (PDF 143 kb)

## ABBREVIATIONS

ALT: Alanine aminotransferase
AST: Aspartate aminotransferase
CMPL: Treatment compliance
Css: Steady state concentration
DFO: Deferoxamine
FT4: Free T4
IIV: Inter-individual variability
IOV: Inter-occasion variability
M&S: Modelling and simulations
NPDE: Normalised predictive distribution error
NTBI: Non-transferrin bound iron
OFV: Objective function value
PKPD: Pharmacokinetic-pharmacodynamic
PRED: Population prediction
RBC: Red blood cells
TSH: Thyroid-stimulating hormone
VPC: Visual predictive check

## References

[1] Gibbons R, Higgs DR, Old JM, Olivieri NF, Swee Lay T, Wood WG. The thalassaemia syndromes. 4th edn. Blackwell Sci; 2001.

[2] Galanello R, Origa R (2010). Beta-thalassemia. Orphanet J Rare Dis.

[3] Ginzburg Y, Rivella S (2011). Β-thalassemia: a model for elucidating the dynamic regulation of ineffective erythropoiesis and iron metabolism. Blood.

[4] Rebulla P (1995). Blood transfusion in beta thalassaemia major. Transfus Med.

[5] Rebulla P, Modell B (1991). Transfusion requirements and effects in patients with thalassaemia major. Lancet.

[6] Rund D, Rachmilewitz E (2005). Beta-thalassemia. N Engl J Med.

[7] TIF. Guidelines for the clinical management of thalassaemia. 2008. 24308075

[8] Porter J, Huehns E (1987). Transfusion and exchange transfusion in sckle cell anaemias, with particular reference to iron metabolism. Acta Haematol.

[9] Cunningham MJ, Macklin EA, Neufeld EJ, Cohen AR (2004). Complications of beta-thalassemia major in North America. Blood.

[10] Borgna-Pignatti C, Rugolotto S, De Stefano P, Zhao H, Cappellini MD, Del Vecchio G (2004). Survival and complications in patients with thalassemia major treated with transfusion and deferoxamine. Haematologica.

[11] Borgna-Pignatti C, Cappellini MD, De Stefano P, Del Vecchio GC, Forni GL, Gamberini MR (2005). Survival and complications in thalassemia. Ann N Y Acad Sci.

[12] Brittenham GM, Cohen AR, McLaren CE, Martin MB, Griffith PM, Nienhuis AW (1993). Hepatic iron stores and plasma ferritin concentration in patients with sickle cell anemia and thalassemia major. Am J Hematol.

[13] Lipschitz D, Cook J, Finch C (1974). A clinical evaluation of serum ferritin as an inndex of iron stores. N Engl J Med.

[14] Puliyel M, Sposto R, Berdoukas VA, Hofstra TC, Nord A, Carson S (2014). Ferritin trends do not predict changes in total body iron in patients with transfusional iron overload. Am J Hematol.

[15] Cappellini MD, Pattoneri P (2009). Oral iron chelators. Annu Rev Med.

[16] Kwiatkowski JL (2008). Oral iron chelators. Pediatr Clin N Am.

[17] Musallam KM, Taher AT (2011). Iron chelation therapy for transfusional iron overload: a swift evolution. Hemoglobin.

[18] Shander A, Sweeney J (2009). Overview of current treatment regimens in iron chelation therapy. US Hematol.

[19] Fisher S, Brunskill S, Doree C, Gooding S, Chowdhury O, Roberts D. Desferrioxamine mesylate for managing transfusional iron overload in people with transfusion-dependent thalassaemia (Review). Cochrane Libr. 2013;(8). 10.1002/14651858.CD004450.pub3 PMC11491190 23963793

[20] Brittenham G, Griffith P, Nienhuis A, McLaren C, Young N, Tucker E (1994). Efficacy of deferoxamine in preventing complications of iron overload in patients with thalassemia major. N Engl J Med.

[21] Novartis Pharmaceuticals UK. Deferoxamine summary of product characteristics. Available from: http://www.medicines.org.uk/emc/medicine/2666.

[22] Porter J (2001). Deferoxamine pharmacokinetics. Semin Hematol.

[23] Theil EC (2009). Mining ferritin iron: 2 pathways. Blood.

[24] Bentur Y, Koren G, Tesoro A, Carley H, Olivieri N, Freedman MH (1990). Comparison of deferoxamine pharmacokinetics between asymptomatic thalassemic children and those exhibiting severe neurotoxicity. Clin Pharmacol Ther.

[25] Porter JB, Rafique R, Srichairatanakool S, Davis BA, Shah FT, Hair T (2005). Recent insights into interactions of deferoxamine with cellular and plasma iron pools: implications for clinical use. Ann N Y Acad Sci.

[26] R Core Team. R: a language and environment for statistical computing. 2014.

[27] Comets E, Brendel K, Mentré F (2008). Computing normalised prediction distribution errors to evaluate nonlinear mixed-effect models: the npde add-on package for R. Comput Methods Prog Biomed.

[28] Hooker AC, Staatz CE, Karlsson MO (2007). Conditional weighted residuals (CWRES): a model diagnostic for the FOCE method. Pharm Res.

[29] Lindbom L, Ribbing J, Jonsson EN (2004). Perl-speaks-NONMEM (PsN)--a Perl module for NONMEM related programming. Comput Methods Prog Biomed.

[30] Gabutti V, Piga A (1996). Results of long-term iron-chelating therapy. Acta Haematol.

[31] Galanello R, Kattamis A, Piga A, Fischer R, Leoni G, Ladis V (2006). A prospective randomized controlled trial on the safety and efficacy of alternating deferoxamine and deferiprone in the treatment of iron overload in patients with thalassemia. Haematologica.

[32] Olivieri N, Nathan D, MacMillan J, Wayne A, Liu P, McGee A (1994). Survival in medically treated patients with homozygous β-thalassemia. N Engl J Med.

[33] Modell B, Khan M, Darlison M (2000). Survival in β-thalassaemia major in the UK: data from the UK Thalassaemia Register. Lancet.

[34] MathWorks T (2010). MATLAB and statistics toolbox release 2010b.

[35] Shampine L, Reichelt M (1997). The MATLAB ODE suite. SIAM J Sci Comput.

[36] Shampine L, Reichelt M, Kierzenka J (1999). Solving index-1 DAEs in MATLAB and simulink. SIAM Rev.

[37] Kattamis A, Dinopoulos A, Ladis V, Berdousi H, Kattamis C (2001). Variations of ferritin levels over a period of 15 years as a compliance chelation index in thalassemic patients. Am J Hematol.

