# Supplementary material for: Model-Based Optimisation of Deferoxamine Chelation Therapy
Source: Pharm Res. 2015 Nov 10;33:498–509. doi: 10.1007/s11095-015-1805-0 (PMC4709373; doi:10.1007/s11095-015-1805-0)

Figure 1S: Goodness-of-fit plots of the model without the parameterisation of compliance. Upper panels show the observed data (Obs) vs. population predictions (Pred) (left) and the observed data vs. individual predictions (IPred) (right). Lower panels show the conditional weighted residuals (CWRES) vs. population predictions (left) and the CWRES vs. time (left).

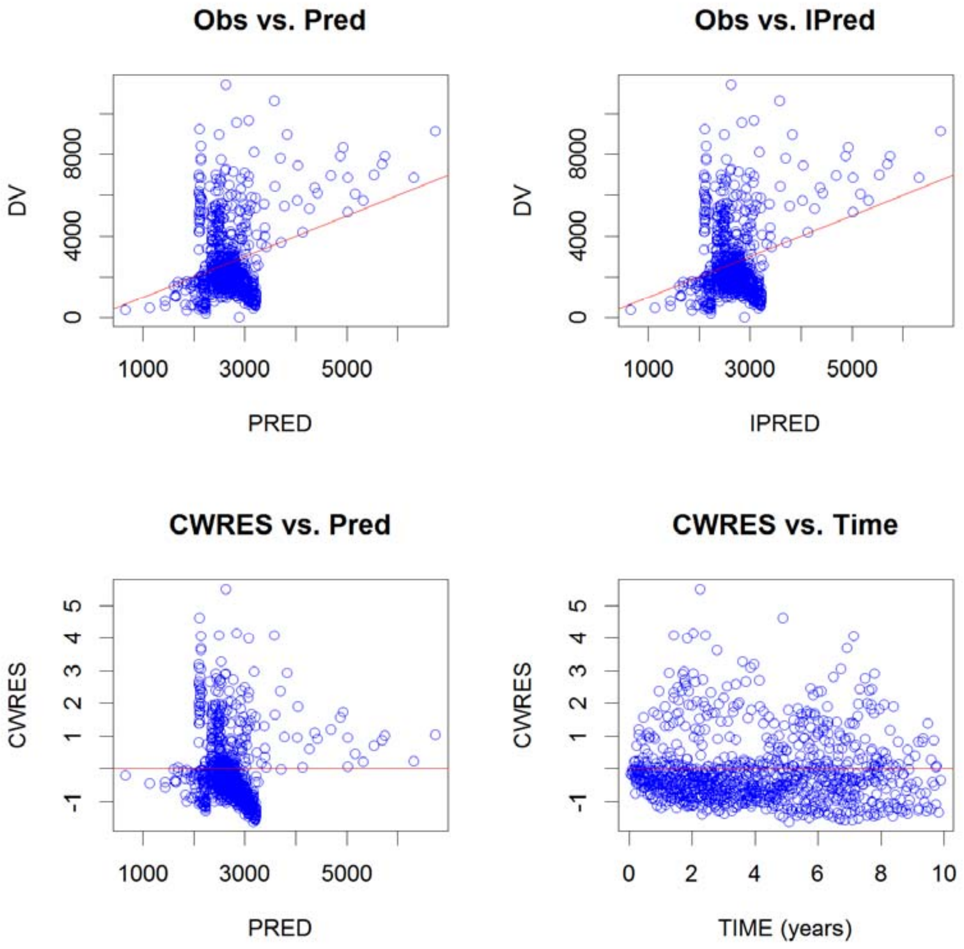

Supplement: Supplementary file 1 — (PDF 140 kb) [file 11095_2015_1805_MOESM1_ESM.pdf]
