# Supplementary material for: Model-Based Optimisation of Deferoxamine Chelation Therapy
Source: Pharm Res. 2015 Nov 10;33:498–509. doi: 10.1007/s11095-015-1805-0 (PMC4709373; doi:10.1007/s11095-015-1805-0)

Figure 2S: Goodness-of-fit plots of the final model, in which compliance was parameterised as a covariate factor. Upper panels show the observed data (Obs) vs. population predictions (Pred) (left) and the observed data vs. individual predictions (IPred) (right). Lower panels show the conditional weighted residuals (CWRES) vs. population predictions (left) and the CWRES vs. time (left).

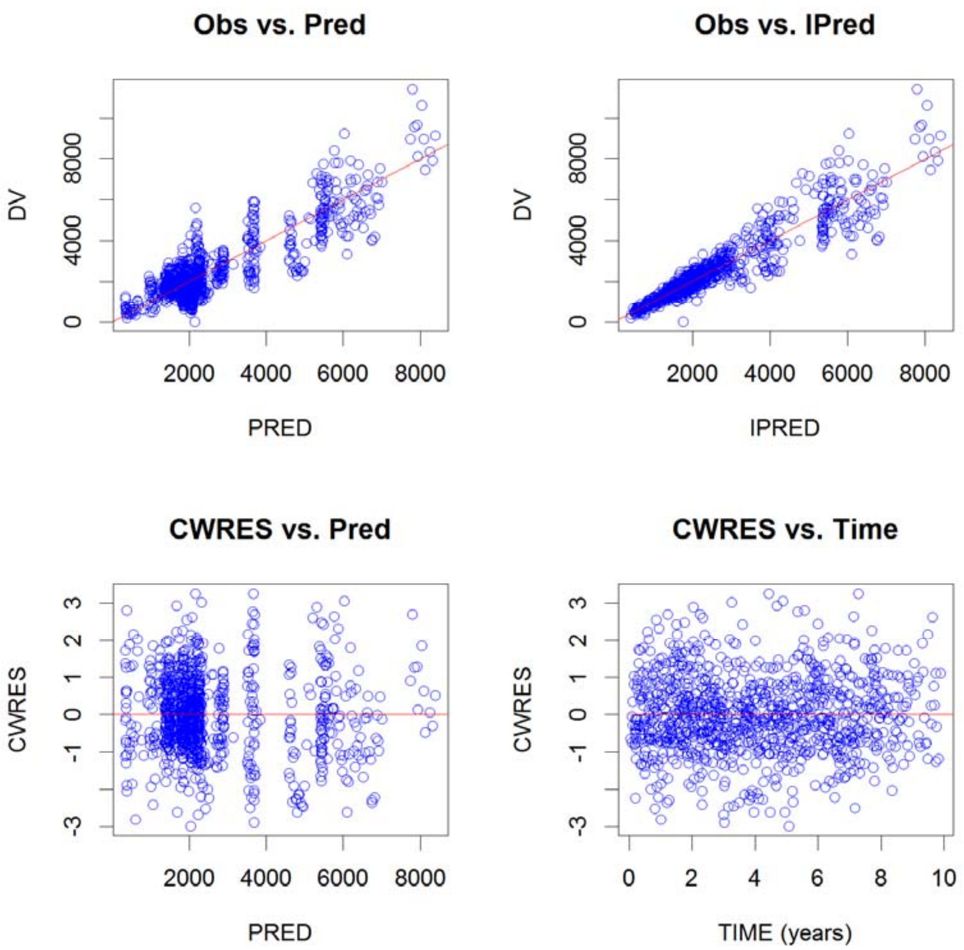

Supplement: Supplementary file 2 — (PDF 152 kb) [file 11095_2015_1805_MOESM2_ESM.pdf]
