# Supplementary material for: Model-Based Optimisation of Deferoxamine Chelation Therapy
Source: Pharm Res. 2015 Nov 10;33:498–509. doi: 10.1007/s11095-015-1805-0 (PMC4709373; doi:10.1007/s11095-015-1805-0)

**Figure 3S:** NPDE: normalised prediction distribution errors. Upper panels show the QQ-plot of the distribution of the NPDEs for a theoretical N (0, 1) distribution (left) and the histogram of the distribution of the NPDE together with the density of the standard normal distribution (right). Lower panels show the NPDEs vs. time (left) and NPDEs vs. individual predictions (right).

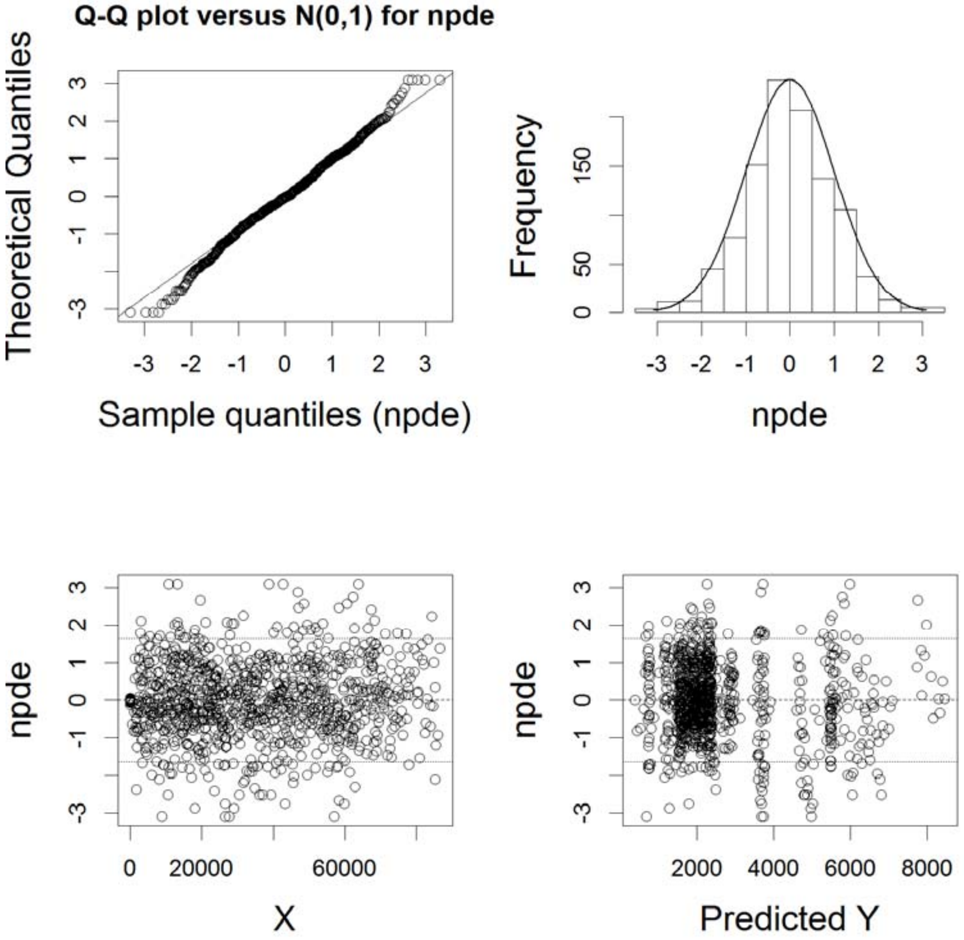

Supplement: Supplementary file 3 — (PDF 143 kb) [file 11095_2015_1805_MOESM3_ESM.pdf]
